# Postoperative apnea after inguinal hernia repair in formerly premature infants: impacts of gestational age, postconceptional age and comorbidities

**DOI:** 10.1007/s00383-013-3330-8

**Published:** 2013-06-19

**Authors:** Tunç Özdemir, Ahmet Arıkan

**Affiliations:** Department of Pediatric Surgery, Tepecik Training and Research Hospital, Gaziler Cd., Izmir, Turkey

**Keywords:** Premature, Inguinal hernia, Apnea

## Abstract

**Purpose:**

It is common practice for premature infants undergoing elective inguinal hernia (IH) repair to be hospitalized for postoperative apnea monitoring. This study evaluated the risk of apnea after IH repair with regard to gestational age (GA) and postconceptional age (PCA) in formerly premature infants.

**Methods:**

Formerly premature infants who had undergone elective IH repair between 01/2000 and 12/2012 were reviewed retrospectively in terms of GA, PCA, body weight, and comorbidities. All postoperative apneas were evaluated.

**Results:**

A total of 428 formerly premature infant charts were reviewed. Eleven babies had postoperative apnea. Infants younger than 45 weeks PCA were found more prone to develop postoperative apnea after IH repair. In older infants (PCA between 46 and 60 weeks), comorbidities create predisposition to apnea postoperatively. These comorbidities are bronchopulmonary dysplasia, necrotizing enterocolitis and former apnea episodes. Anemia and lower birth weight are also risk factors.

**Conclusion:**

This study suggests that low GA and PCA, low birth weight, anemia, and complicated past medical history affect respiratory complication rates, particularly apnea in formerly premature infants undergoing elective IH repair. Severe apneas occurred earlier than mild ones. Overnight monitoring is mandatory in small infants with low GA and PCA. Otherwise healthy, older infants may be operated on outpatient basis.

## Introduction

Formerly premature infants undergoing herniorraphies were more prone to postoperative respiratory complications as compared with full-term infants. The incidence of respiratory complications is reported as 33 %, with apnea most common among premature infants after herniorraphy [1]; the incidence for full-term infants was 2.6 %.

There is no agreement about the optimal time to repair the asymptomatic inguinal hernia (IH) discovered in a premature infant. In prematures with a known IH, it is still debated whether to perform repair before discharge from the neonatal intensive care unit (NICU). Some advocate repair to prevent the risk of incarceration, whereas others recommend repair as an elective procedure to minimize anesthetic risks.

It is also common practice for premature and formerly premature infants undergoing elective IH repair to be hospitalized for possible postoperative apnea.

This study is designed to determine whether it is necessary for all former premature infants undergoing elective herniorraphy to require overnight hospitalization, or a 6- to 12-h postoperative observation period instead.

## Methods

All formerly premature infants with a postconceptional age (PCA) less than 60 weeks, having herniorraphy and admitted to the ICU for apnea monitoring postoperatively between January 2000 and December 2012, were included in this study. All patients had blood draws for hemoglobin level before elective IH repair.

Anesthesia was commenced with an inhalational induction with oxygen/air and up to 8 % inspired sevoflurane (induction dose 5–8 %, maintenance 0.5–3 %). Maintenance of anesthesia was obtained with laryngeal mask. Herniorraphies were performed by three senior surgeons and the type of surgical repair performed was constant throughout the series. Surgical time varied between 8 and 15 min (mean 10 ± 0.5 min). Postoperative analgesia was achieved with rectal paracetamol.

The standard protocol for overnight hospitalization includes patients born before 36 weeks gestation who are less than 60 weeks PCA. All patients had continuous cardiorespiratory monitoring postoperatively throughout their hospital stay. An apneic event was counted by either documentation of apnea by nursing notes through visual observation or documented pauses ≥15 s. Severe apnea was defined as a desaturation of ≤85 % or requiring mask assistance for apnea.

The principal outcome variables considered are, gestational age, birth weight, medical history, and hemoglobin level at time of surgery, PCA at surgery, weight at surgery, pre- and postoperative respiratory complications and the length of hospital stay.

Patients were divided into two groups for data analysis. Group 1 was composed of patients less than 45 weeks PCA, group 2 was between 45 and 60 weeks PCA to define the age group at greatest risk of postoperative apnea. Comparison among groups was performed using analysis of variance for continuous variables and *x* ^2^ for categorical variables. Significance was defined as *p* < 0.05.

## Results

A total of 428 formerly premature infant charts was reviewed: 351 males and 77 females. There were 191 patients in group 1 and 237 in group 2. Table 1 summarizes the demographic data and associated comorbidities of two groups. Eleven babies had postoperative apnea. Nine of these patients who suffered from apnea were from group 1 (9 of 191, 4.7 %). The other two patients were from group 2 (2 of 237, 0.8 %) (Table 2). The difference between two groups regarding the occurrence of apnea was statistically significant (*p* < 0.05).

**Table 1:** Comorbidites and demographic data of the patients in both groups

	Group 1	Group 2
Total no. of patients	191	237
GA (week)	30 ± 3.5	31 ± 4
PCA (week)	42 ± 4	53 ± 7
Birth weight (g)	1,600 ± 660	1,550 ± 700
History of mechanical ventilation	38 %	39 %
Associated cardiac anomaly	21 %	20 %
BPD	17 %	19 %
NEC	1.2 %	1 %
Hemoglobin level (g/dl)	9.6 ± 2	9.2 ± 1.5

*BPD* bronchopulmonary dysplasia, *NEC* necrotizing enterocolitis

**Table 2:** Cross tabulation of the apnea groups

	Apnea		Total
	Apnea (−)	Apnea (+)	
Group			
G1			
Count	182	9	191
% Within group	95.3	4.7	100.0
% Within apnea	43.6	81.8	44.6
G2			
Count	235	2	237
% Within group	99.2	0.8	100.0
% Within apnea	56.4	18.2	55.4
Total			
Count	417	11	428
% Within group	97.4	2.6	100.0
% Within apnea	100.0	100.0	100.0

The oldest GA for any of these infants was 32 weeks and oldest PCA was 56 weeks. Bag and mask ventilation was required in four cases, and three cases required stimulation. One apnea was self-terminating. Three infants suffered from severe apnea and required intubation and mechanical ventilation for a definite period (mean 3 days, between 1 and 7 days).

In seven patients, apnea occurred at a mean 4 h postoperatively with a range of 1–11 h. Other four patients’ apneas occurred at a mean of 17 h postoperatively with a range of 8–22 h.

Regarding the groups, mean apnea time was found 8.6 h (2–18 h) for group 1. Mean apnea time was 4 h (2–6 h) in group 2.

Mean birth weight was slightly lower in group 2 than group 1 (1,300, 1,555 g respectively, *p* < 0.05). Although infants from group 1 were older from group 2, mean weight at the time of operation was not significantly different (3,338 g in group 1, 3,455 g in group 2, *p* > 0.05).

In infants who suffered from apnea, no difference was encountered in terms of GA among groups (in group 1, 28.1 weeks; in group 2, 28.5 weeks, *p* > 0.05).

All three infants requiring intubation and mechanical ventilation were the patients from group 1. And all these patients’ birth weight was below 2,000 g (100 %, *p* < 0.05). We found several strong predictors of apnea from the patient’s past medical history in group 2 (Table 2). Previous history and BPD and NEC were identified as significant risk factors. Two patients from group 2 who suffered apnea had severe comorbidities previously (100 %, *p* < 0.05). Previous history of BPD was a strong predisposing factor in patients of group 2. No other patients suffered from apnea in group 2 without comorbidities. Severe apneas occurred within 4 h postoperatively. No other patients required intubation and mechanical ventilation beyond 4 h after surgery. One of the nine infants from group 1 was anemic at the time of operation.

## Discussion

Despite being one of the most common procedures performed by pediatric surgeons, many questions still persist with respect to optimal timing for inguinal herniorraphy in former premature infants. Complications after IH repair occur in 1.7–8 % of all cases, although these studies vary in their reporting of “minor complications” and generally include pediatric patients of all ages [2–4].

Prematures and formerly premature infants undergoing herniorraphies are more prone to postoperative respiratory complications, particularly apnea, as compared with full-term infants. The initial documentation of postoperative apnea in former premature infants recovering from general anesthesia appeared in 1982 [1]. Apnea of prematurity and postanesthetic apnea appear to bear no relationship to sudden infant death syndrome. General anesthesia, however, can induce or unmask abnormalities or ventilator control not previously noted in a former preterm or even a term infant [1]. The reported risk of postoperative apnea is as high as 49 %, while more recent reports demonstrated this rate to be closer to 5 % [5–8]. The 1995 meta-analysis of eight small series by Cote et al. [5] used the widely variable incidences reported in these series to establish a predictive curve, which pointed to a significant reduction in the incidence of apnea at 52–54 weeks PCA, with an incidence of apnea to less than 1 % at 54 weeks PCA. They determined that (1) apnea was strongly and inversely related to both GA and PCA; (2) an associated risk factor was continuing apnea at home; (3) small-for-gestational-age infants seemed to be somewhat protected from apnea as compared to appropriate- and large-for-gestational-age infants; (4) anemia was a significant risk factor, particularly for patients >43 weeks’ PCA; (5) a relationship to apnea with previous comorbidities or operative use of certain drugs could not be demonstrated [5]. Anemia seemed an important factor yielding to postoperative apnea in our series as well. The risk of apnea is known to decrease with increasing PCA and GA at birth. Although PCA is probably the most important characteristic in identifying the high-risk patient, it is difficult to determine a minimum PCA that is associated with least post-anesthetic respiratory complications particularly apnea. Additionally, associating comorbidities must be taken into account to determine the risk groups.

A study by Abajian et al. [9] in 1984 sparked an interest in spinal anesthesia as an alternative to general anesthesia in this high-risk population. Since that time, numerous other studies have been published examining the risk of apnea in preterm infants after receiving spinal anesthesia [10–12]. Veverka et al. [13] described 83 preterm, high-risk patients who underwent IH repair with spinal anesthesia with no episodes of apnea. Frumiento and Abajian [10] reported that patients with a previous history of apnea are at increased risk for postoperative apnea after spinal anesthesia. A study comparing spinal and general anesthesia showed that spinal anesthesia without ketamine sedation was not associated with postoperative apnea, whereas infants receiving either general anesthesia or spinal anesthesia plus ketamine sedation experienced a 31 and 89 % incidence of postoperative apnea, respectively [14].

Lee et al. [15] suggested that there was minimal risk of postoperative apnea for premature infants undergoing elective inguinal hernia repair, thus they are questioning whether routine hospital admission is required.

It has been shown that the older the PCA, the lower the risk of postoperative apnea [5]. A meta-analysis has shown that all former premature infants with PCA less than 46 weeks be observed for at least 12 h, with individualized patient care based on associated comorbidities for those between 46 and 60 weeks [16]. The authors recommend administration of intravenous caffeine (10 mg/kg) to reduce the risk of apnea [16]. A more recent study suggests overnight observation for patients born before 37 weeks gestation who are under 50 weeks PCA [17]. However, a lack of consensus on the definitions of apnea and bradycardia creates a concerning source of bias in previous studies.

In this study, the overall risk of postoperative apnea was 2.5 % in formerly premature infants. This rate is elevated to 4.7 % in group 1, which consisted of infants with PCA less than 45 weeks. In infants older than 45 weeks of PCA, the rate is 0.8 %. Low birth weight is also a risk factor. However, comorbidities seem influential for postoperative apnea in group 2. Comorbidities that have shown to increase the risk of postoperative apnea are BPD and NEC for this group. No patients without comorbidities suffered from postoperative apnea in group 2. These data reveal that PCA was the primary determinant of postoperative apnea. Additionally, severe apnea requiring intubation and mechanical ventilation occurred mainly in patients from group 1. These patients suffered from apnea earlier than other patients as well.

Although, mean birth weight was slightly higher in group 2 with regard to weight at the time of operation, mean weight was not significantly different. This may be due to comorbidities of the two patients from group 2 who suffered from apnea (BPD in one infant, BPD and NEC in other one). It also may be speculated that previous comorbidities causing growth retardation might become a predisposing factor for postoperative apnea.

Gestational age was an important variable in the apneic group (*p* > 0.05). This may be due to the low number of apneic patients in group 2. This variable may be analyzed by a prospective study in the future.

In conclusion, PCA is the most important variable predicting the probability of apnea in formerly premature infants. We found 4.7 % rate of apnea in this subset of infants, which may require intubation and mechanical ventilation. This risk may persist beyond 12–18 h. Therefore, infants with PCA younger than 45 weeks would need overnight monitoring. Similarly, regardless of PCA, previous comorbidities like BPD, NEC history of apnea are at great risk of postoperative apnea after IH repair. Although the number of patients is low for this subset of patients (*n*: 2), it may be concluded that, these patients would not need monitoring beyond 6 h. Formerly premature infants with PCA older than 45 weeks and without comorbidities may undergo IH repair at outpatient basis. The otherwise healthy infant could be scheduled for theater as the first patient on the list and subsequently monitored in the post-anesthetic care unit for 6 h. As we found anemia seems a causative factor for postoperative apnea, hemoglobin level should be assessed preoperatively in formerly premature infants who would undergo IH repair.

There are several important limitations to consider in evaluating the data generated by our study. Because the study is retrospective in nature, data collected were not expressly generated for research and are, therefore, subject to all of the inherent biases of such methodology. Relative to several other investigations of apnea in formerly premature infants who had undergone IH repair, this study is large and derived from experience of a single institution. Our clinical practice on formerly premature infants has changed after multifactor analysis of this study according the conclusion above.
